# Advancing public health policy making through research on the political strategies of alcohol industry actors

**DOI:** 10.1093/pubmed/fdz031

**Published:** 2019-04-09

**Authors:** Jim McCambridge, Kypros Kypri, Trevor A Sheldon, Mary Madden, Thomas F Babor

**Affiliations:** 1 Department of Health Sciences, Seebohm Rowntree Building, University of York, Heslington, York YO10 5DD, UK; 2 School of Medicine & Public Health, University of Newcastle, Australia; 3 Department of Community Medicine and Health Care, UConn Health, Farmington, Connecticut

**Keywords:** alcohol, public health

## Abstract

Development and implementation of evidence-based policies is needed in order to ameliorate the rising toll of non-communicable diseases (NCDs). Alcohol is a key cause of the mortality burden and alcohol policies are under-developed. This is due in part to the global influence of the alcohol industry. We propose that a better understanding of the methods and the effectiveness of alcohol industry influence on public health policies will support efforts to combat such influence, and advance global health. Many of the issues on the research agenda we propose will inform, and be informed by, research into the political influence of other commercial actors.

Alcohol was a cause of ~3 million deaths in 2016, and is the leading risk factor for mortality among people aged 15–49 years worldwide, as well as being responsible for extensive social problems.^1^ Effective alcohol policies are urgently needed.^2^ There is growing evidence that involvement of alcohol industry actors in policy making results in decisions that favour commercial interests over public health.^3^ Alcohol policy lags behind other areas globally, e.g. on obesity or tobacco control, where high level political commitments have already been made, and resourcing and implementation of these commitments at the national level are the key issues to be faced.^4^ Controversies about the relationships between alcohol industry and public health actors, such as the recent Drinkaware—Public Health England partnership, to the detriment of public health^5^ appear to be perennial. We argue in this ‘Perspectives’ paper that such episodes occur because the public health community understands too little about alcohol industry political strategies.

## The elephant in the room

There have been warnings about the adverse consequences of alcohol industry activities for more than a quarter of a century.^6^ Whilst evidence-informed national alcohol policies are recommended by the World Health Organization (WHO),^2^ they are resisted by the alcohol industry.^7^ A WHO survey of national alcohol policies identified interference by the alcohol industry as a key obstacle,^8^ particularly in relation to strategies known to be effective in yielding population-level reductions in the harmful use of alcohol.^9^ The alcohol industry is ‘the elephant in the room’, and an obvious problem that few have been willing to discuss,^10^ but this has begun to change.^3,11–19^ Recent research attention to the alcohol industry mirrors the growth of public health research devoted to corporate actors in other sectors,^20–22^ for which the Lancet NCD Action Group recommended the development of ‘a new scientific discipline that investigates industrial diseases and the transnational corporations that drive them’^20^.

## The basic features of the elephant: what do we already know?

Here we take stock of the emerging literature. The alcohol industry is organized within beverage classes and includes the supply chain,^11^ increasingly concentrated in a small number of transnational corporations. These companies appear to have altered the political involvement of industry trade associations, and developed social aspect/public relations organizations.^11,15^ The latter claim to be concerned with reducing the social harms caused by alcohol as a form of corporate social responsibility (CSR) but actually serve public relations functions for the alcohol industry, framing policy issues in ways which serve industry interests.^23^ Industry actors, whether they be companies or other types of organization, seek to work in partnership with national governments, and where possible, have direct involvement in decision-making within public health policy making.^24^ Evidence suggests industry political strategies are flexibly and pragmatically adapted to different policy venues and institutions, the place of alcohol within the culture, and wider aspects of the political climate.^3,25–27^

Industry actors routinely claim that they are committed to evidence-informed policy, seeking partnerships with governments, yet to avoid decreasing profits they misrepresent scientific evidence and oppose regulatory approaches that scientific evidence shows are most likely to be effective.^28^ They instead argue for self-regulation, for which there is little supporting evidence.^19,29^ In the UK, the extent to which public health evidence has been used in policy making may help explain the divergence between England and Scotland.^27,30,31^ There are also now studies of particular social aspect/public relations organizations,^12,19,32,33^ as well as examination of particular campaigns^34^ and relationships with charities.^35,36^ Social aspects organizations produce or fund books, reports and policy briefs which provide industry-favourable and biased views of the evidence, distort ideas about policy options, and recommend policies unlikely to be effective.^12,19,37^ Funding think tanks and other third parties also appears important in commercial actor approaches to influencing alcohol policy making.^3,36,38,39^ Third party reports provide a parallel literature to the international, peer-reviewed scientific literature that industry actors use to support their policy positions.^12,38^

When partnership approaches with national governments fail to influence policy, confrontational tactics may be employed. The legality of the Scottish Government’s minimum unit pricing measure, for example, was challenged by industry actors, who successfully delayed implementation by ~6 years,^27^ in ways similar to the tobacco industry’s efforts to thwart public health policies.^40^ International trade law has also been used by some national governments to oppose the national alcohol policies of other countries to further the interests of their national alcohol industries.^41^

## Similarities with other commercial sectors?

This brief overview of what is known about the alcohol industry may resonate with perceptions of what powerful corporations do more broadly. There has been little systematic study, however, of industry strategies and tactics across sectors that produce and market health damaging commodities, and a concomitant lack of attention to the development of theories to underpin research agendas. The exercise of policy influence by powerful corporate actors is a complex and contested object of study.^22^ Earlier investigations of the tobacco industry were greatly advanced by access to internal company documents, made available following litigation, providing a wealth of information about tobacco industry political strategies.^42^ These documents suggest important implications of cross-ownership between tobacco and alcohol companies,^12,43–45^ and identify evidence of common strategies between the two industries to influence tobacco control policy.

Ulucanlar and colleagues^46^ have conceptualized tobacco industry political activities in terms of narratives articulated to advance policy preferences in combination with direct involvements in policy making. The latter include coalition management (of allies with similar interests) and information management (having well developed and timely supportive content available, e.g. policy briefings).^46^ A systematic review identified many similarities between tobacco and alcohol industry political activity in respect of attempts to influence policy on marketing, with differences likely due to varying policy contexts and access to decision makers.^47^ Another systematic review of alcohol industry involvement in policy making more broadly, drew similar conclusions.^3^ This may be because there are similar strategic challenges facing both industries in dealing with public health policy makers, stemming from the fact that both products cause addiction and other health harms,^22,48,49^ and are often used together. The intoxicant effects of alcohol, and the resulting harms to self and others, pose additional strategic challenges. The closer the observed relationships between tobacco and alcohol industry actors in addressing common challenges, the more research on the alcohol industry can be informed by the extensive knowledge available on the tobacco industry.

In addition to the apparent similarities and overlaps between the alcohol and tobacco industries, alcohol industry actors are also linked to other corporate sectors in numerous ways. Freudenberg draws attention to financial institutions, advertising agencies, law firms, lobbyists, politicians, scientists and journalists as comprising a ‘corporate consumption complex’ that supports corporate sectors such as alcohol and ultimately threatens public health.^50^

The internal composition of, and political dynamics within, the alcohol industry may be distinct from other sectors, and there has been little previous research attention to inter-relationships between corporate sectors other than with tobacco. The major alcohol producers have expanded their holdings in the soft drink market, where there is growing evidence of the health hazards of sugar-sweetened beverages, and consumers may be becoming more health conscious.^51^ Alcohol industry actors use CSR to frame key issues for the general public health and policy actors,^23^ and it has been suggested that CSR may also be designed to support the marketing of potentially harmful products.^51^ It is worth investigating whether CSR may be more integral to political and/or market strategy development among alcohol companies than other corporate sectors.

Nationally based supermarkets may be better placed to influence national policies than transnational producers.^52^ Alcohol industry and other corporate interests have varied forms of political organization, priorities and positioning in different countries, so it should be expected that political practices will need to be investigated in dedicated studies at the national level (as well as at other levels of governance, such as the local [community], regional [e.g. European Union] and global [e.g. United Nations/WHO]).

## What kinds of research are needed?

Contemporary studies of tobacco, food and other commercial actors relevant to public health rely extensively on documentary analyses of publicly available data. Data sources may include annual reports, communications to shareholders or investors, responses to government consultations, material published on company websites and social media, media coverage of corporate affairs, market intelligence reports, official reports, freedom of information requests and litigation records.^53^ The use of such data sources has made limited contributions to the study of the alcohol industry to date, with the possible exception of analyses of public consultation responses.^28,54–56^

One theoretical approach developed in the alcohol research community is the epidemiologic cascade model, which identifies corporations as ‘upstream’ inducers of disease^15,57,58^ due to influence on government policy, which in turn makes for a more alcogenic environment that results in increased drinking of their products and non-communicable diseases (NCDs). This perspective has been influential in wider public health, with similar multi-level cascade models having been applied to corporate determinants of health in other sectors.^59–62^

Better understanding of the methods and the effectiveness of commercial actor influence on public health policies makes it possible to study ways to combat such influence. Moving from what might be characterized as an observational to an intervention research perspective may take advantage of dedicated initiatives by policy makers, NGOs and the public health community more generally, which may offer possibilities for study as natural experiments to better manage commercial actor political strategies.^63^

Alcohol industry opposition to the directions in research advocated in this paper should be expected, as was the case when industry pressure recently succeeded in halting a study of warning labels being undertaken in government liquor stores in the Yukon, Canada.^64^ To date, there has been limited study of alcohol industry involvement in science,^65^ despite longstanding grounds for concern and the strategic significance of scientific research for the tobacco companies.^13,66^

Several large producers and trade associations have recently invested significant resources in large-sale research projects which themselves warrant evaluations of scientific integrity, and their use for framing purposes in advancing CSR and commercial agendas. MACH15 was a $100 million, nine-nation clinical trial mostly funded by major producers to evaluate the health benefits of a single dose of alcohol given daily to persons at risk of coronary heart disease.^67^ A controversy ensued after the trial began, which precipitated a NIH investigation, ultimately terminating the trial.^68^ Other major research initiatives include the ABInBev Global Smart Drinking Goals programme which includes funding an external evaluation of an alcohol intervention toolkit that is being implemented in ‘pilot’ cities located in six countries.^69^

Given the need to study the political activities of commercial actors, there are important contributions to be made by social science disciplines, in tandem with public health sciences. It is noteworthy that the literature cited to date largely originates from the latter. The importance of issue salience with the public has been emphasized by political scientists as a key determinant of corporate capacity to shape policy outcomes.^70^ This ‘quiet politics’ framework for analysing policy suggests that when and where there is little media and public discussion of alcohol and alcohol problems, industry actors are more able to exercise influence over policy. This in turn shapes both the framing of which problems policy makers may be concerned with, and of possible solutions to policy issues. There is now research on misleading claims made by industry actors on cancer directed at the general population,^71^ public understanding of the activities of alcohol industry organizations,^72^ and other work on alcohol industry framing of media content.^34,73^ Such research, which serves to disrupt the efforts by industry actors to keep the politics quiet, may thus be particularly valuable to study.

Interview and ethnographic studies are needed to complement research using public domain data because much policy influence is designed to be invisible, meaning that reliance on public domain data alone would be of limited value. Social sciences informed interview studies have been used to better understand the nature of the political activities of alcohol industry actors.^30,52,74–76^ So far as we are aware there have been no studies of, for example, large alcohol companies’ use of their financial resources, contract lobbyists, the functions and operation of trade associations, or revolving doors personnel movements between corporate and public sectors anywhere in the world. The wider literature on lobbying suggests that these drive key asymmetries in information, personnel and other resources that allow corporate actors’ influence in policy making.^77^ To make progress in the direction proposed here requires dedicated research funding as an investment in developing public policy and protecting it from vested interests. Dedicated funding may be challenging to obtain, however, until there is greater awareness of the need to address this major societal challenge. Researchers should, in the meantime, strive to build the evidence-base, even in the absence of dedicated funding, to draw attention to this situation.^78^

In Box 1 we offer examples of the sorts of research questions that could be valuable to investigate, based on what is known about the alcohol industry. Wider research community, and indeed public health policy actor, involvement in developing research agendas will accelerate progress and priority setting exercises may be useful. Note that few of these questions apply only to alcohol and alcohol industry actors. Our intent is not to be prescriptive but rather to stimulate creative thinking about possible ways forward.

Box 1. Sample research questions

**Table:** 

*How much and in which ways does the history and culture of alcohol in a given country shape consideration of national alcohol policy?* *How do industry actors use CSR, marketing and other means to shape the way policy-makers and the public view alcohol problems and possible policy responses?* *Which are the key alcohol industry actors to study in respect of policy influence?* *How similar and different are the strategies of alcohol industry actors to those of tobacco, food, gambling and other unhealthy commodity industry actors?* *Does strong public opinion on policy issues reduce the degree of unhealthy commodity industry activity concerning those issues?* *How do global unhealthy commodity industry actors adapt their strategies to national-level political institutions?* *What strategies do unhealthy commodity industry actors use to ‘quieten’ the politics concerning their products and activities?* *What are the roles of contract lobbyists, think tanks and other third party actors in shaping relationships with policy-makers?* *What can public registers and freedom of information requests reveal about the scale and patterns of gifts and donations to political parties, and about lobbying?* *What is the extent and pattern of employing former politicians and civil servants in government relations and similar roles in unhealthy commodity industry companies?* *Are relationships forged between industry actors and government officials in similar ways in low- and middle-income countries compared to high income countries?* *What countermeasures to unhealthy commodity industry influence are available in high, middle, and low income countries?* *How do supra-national policy processes influence national-level policy making?*

## The stakes are high

The accelerating concentration of beer and spirits producers into a small number of transnational corporations makes the structure of the alcohol industry increasingly resemble the tobacco industry, dominated by a small number of global producers.^49^ The merger of the two largest brewers in the world (ABInBev’s acquisition of SAB Miller) means a single company now produces approximately one-third of all beer sold globally, a higher market share than possessed by any one tobacco company.^49^ The core rationale for this merger was to develop markets in Africa.^79^ Alcohol market expansion entails harmful consumption of alcohol becoming more prevalent.^80^ Addressing harmful drinking is a key target of the Sustainable Development Goals (SDG).^63^ Unless the political influence of the alcohol industry is effectively constrained, it is unlikely that per capita consumption (SDG indicator 3.5.2) will be reduced in many low and middle income countries and the global target achieved.^18,81^

According to former WHO Director, Margaret Chan, alcohol industry actors should not be involved in alcohol policy development.^7^ Nevertheless, WHO is planning to engage with the private sector, including ‘economic operators in alcohol production and trade—to contribute to reducing the harmful use of alcohol, including labelling, marketing and retail sales practices’.^82^ The public health community, including WHO, needs to better understand the risks intrinsic to such engagements, develop strong procedures for the management of the inherent conflicts of interest, and study how industry actors use such engagements to their advantage. There is also a need to develop a narrative on reasonable expectations of alcohol industry actors in the public interest (see Babor *et al.*^83^ for an example). WHO’s own position that engagement should be restricted to minimizing the harms of their products and marketing is a particularly important guiding principle.^84^

The elephant metaphor we have used would not be complete without reference to the parable of the Seven Blind Men of Hindustan, who encounter a large animal that each one attempts to describe from the limited perspective of the body part that they have been able to touch. The moral of the story is that when observers of the same phenomenon project their partial experience into their interpretation of a larger reality, they misunderstand the nature of the beast. Researchers, similarly, often seek to explain alcohol problems or other causes of NCDs by examining different aspects of the industry, such as the type of product, marketing, price or availability, the amount consumed by an individual or a population, or its effects on a small number of excessive users, without recognizing that these are all parts of a larger whole. We suggest a useful heuristic approach to explaining the contemporary nature of alcohol’s contribution to NCDs is to first view the global expansion of the alcohol industry and its concentration in a small number of global producers as needing to be understood structurally. Secondly, its increasing use of sophisticated marketing is located within overarching corporate strategies that also incorporate explicitly political strategies designed to create and sustain environments in which markets can be developed.

The lack of a well established body of research evidence on the alcohol industry’s political activities limits the ability of policy makers to develop approaches that protect against the influence of these and other powerful commercial interests. This influence process is critical to study because harmful producers have strong vested interests to obstruct policies that place constraints on their business activities,^57,58^ and are usually but not always successful in so doing.^85^ Strengthening research capacity to investigate the alcohol industry can thus equip public health decision makers to develop effective alcohol policies. It can also advance global research agendas on vested interests and policy making, and contribute more broadly to effective national and global health strategies, by identifying differences as well as similarities in political strategies across corporate sectors. We suggest there is a clear need to generate a step change in alcohol public health policy research, securing funding and undertaking programmes of research, developing new research agendas, and building capacity by developing new public health oriented networks, including those that link this particular sector to other corporate sectors. This should be a global health research priority, and involves expanding the horizons of alcohol-specific and broader public health research.^86^ Such research is needed to advance public health policy.
